# Health-Care Metrics in Oncology

**Published:** 2015-01-01

**Authors:** Elizabeth Gilbert, Victoria Sherry, Suzanne McGettigan, Anne Berkowitz

**Affiliations:** Abramson Cancer Center, University of Pennsylvania, Philadelphia, Pennsylvania

In 1986, the Institute of Medicine (IOM) set out to define health care in terms of quality. Its goal was to establish standards for desired health outcomes that were consistent with current professional knowledge (Donaldson & Lohr, 1990). It has become apparent, however, that variability in the quality of cancer care exists across the United States (Desch, 2008; Hewitt & Simone, 1999; Neuss, 2013).

In the 1999 report of the National Cancer Policy Board (NCPB) entitled "Ensuring Quality Cancer Care," it was concluded that "for many Americans with cancer, there is a wide gulf between what could be construed as the ideal and the reality of their experience with cancer care" (p. 215). Inconsistencies in cancer care were identified, including the lack of patient-focused care, the lack of evidence-based care, and the lack of care coordination (Hewitt & Simone, 1999). Their recommendations included the creation of quality metrics and a national reporting system (Hewitt & Simone, 1999).

Current challenges in health care such as access, cost, quality, and safety have ignited a renewed emphasis on defining and improving health care. The follow-up report by the IOM in 2013, entitled "Delivering High-Quality Cancer Care," identified six specific components to achieving high-quality cancer care. A key component was quality measurement and performance improvement (Levit, Balogh, Nass, & Gantz, 2013). Common pathways to achieve both of these goals are to measure tangible actions, interventions, and processes, collectively known as metrics.

## EVOLUTION OF HEALTH-CARE METRICS

The Oxford dictionary defines *quality* as "the standard of something as measured against other things of a similar kind; the degree of excellence of something." Quality as it applies to health-care delivery has been well described as the "evaluative dimension of the elements and interactions of the medical care process" (Donabedian, 1968). There is often no agreed-upon "standard of excellence," but there are a multitude of agencies that promote standards in patient care by using evidence-based guidelines. The National Quality Forum (NQF) and the Joint Commission are just a few examples (National Quality Forum, 2014; The Joint Commission, 2013 Today, the integration of quality standards with insurance reimbursement for services is becoming more common. It is because of this integration of standards that insurance providers are also driving quality measures in health care.

One of the most influential insurance agencies, the Centers for Medicare and Medicaid Services (CMS), was created in 1965. Medicare is the government-sponsored program that provides insurance coverage for people over the age of 65, those under 65 with certain disabilities, and individuals with end-stage renal disease (CMS, 2013). As of May 2014, more than 54 million people in the United States were insured through Medicare, making it the largest and most powerful health coverage insurer in the United States (Kaiser Family Foundation, 2014). As Medicare moves to require proof of quality through specific measures, it is critical that all health-care providers understand and find ways to meet those standards. The use of metrics is one proposed mechanism to help achieve this goal (Donabedian, 1968).

In Donabedian’s still relevant paper, "Evaluating the quality of medical care," (Donabedian, 1966), he discusses two ways of measuring quality in health care. He discusses the fact that *outcome measures* (i.e., survival data and degree of patient satisfaction) and *process measures* (i.e., applying medical care that is known to be good) can be used, though with limitations, to demonstrate the quality of health-care delivery. Because insurers are starting to use both process and outcome measurements to determine payments to providers, it is even more important to be knowledgeable about their use.

## MEDICARE AND METRICS

Since 2003, CMS started initiatives in paying providers for performance (Kahn et al., 2006). Tracking evidence-based metrics, CMS rewarded providers with monetary bonuses for reaching predetermined standards in quality care. Not only did CMS create positive incentives, but it also instituted penalties for standards not met (Rau, 2011). CMS selects measures based on a wide range of factors, from patient and/or caregiver engagement to conditions that represent national public health priorities (CMS, 2013). Examples of the measures include the eRx Incentive Program, Hospice Quality Reporting Program (HQRP), and Hospital Quality Initiative.

The eRx Incentive Program reports the utilization of medication electronic prescribing by eligible professionals and uses an integration of incentive payments and payment adjustments as motivation. The Hospice Quality Reporting Program requires hospice providers to report quality data such as pain, patient safety, and medication errors to CMS. Failure to meet these requirements results in a two percentage point reduction in the Annual Payment Update. Lastly, the Hospital Quality Initiative compares the quality of care delivered by hospitals and provides these data to the public. The intent of the program is to create incentives to improve hospitals’ care and support public accountability.

Another initiative that came from CMS was the Physician Quality Reporting Initiative (PQRI) in 2007. According to CMS, this program was originally designed as a voluntary reporting program (CMS, 2013). It was developed and implemented as a pay for performance to encourage quality improvement and avoidance of unnecessary costs in the care of Medicare beneficiaries. As of 2010, there were 179 measures. As this program evolved, incentive payments increased, and reporting has become mandatory; the program is now called the Physician Quality Reporting System (PQRS). Beginning in 2015, penalties will be implemented for physicians who opt not to participate (CMS, 2013).

In 2011, CMS released the final rules for the implementation of Affordable Care Organizations (ACOs) under the Affordable Care Act (ACA). The aim of ACOs is to guide health-care providers toward more coordinated, higher-quality, patient-centered care through the use of multiple defined process and outcome measures. A provision of this ACA was the creation of the Patient Centered Outcomes Research Institute (PCORI). Additional provisions of the ACA include nonpayment for the treatment of certain hospital-acquired infections and nonpayment for hospital readmissions (CMS, 2013; Berenson, Paulus, & Kalman, 2012).

## EVOLUTION OF METRICS IN ONCOLOGY

One of the most expensive and increasingly expanding specialties in medicine is the field of oncology. In 2009, the National Institutes of Health estimated that the overall cost of cancer in the United States was $216.6 billion (American Cancer Society, 2014). The American Cancer Society estimates that about 1,665,540 new cancer cases were diagnosed in 2014 and that the lifetime risk of an individual to develop cancer in the United States is 1 in 2 for men and 1 and 3 in women. As the baby boomer population ages, the cost of care is expected to rise exponentially.

Cancer care is increasingly complex and expensive (Meropol et al., 2009). Amplifying the concerns about the cost and the decreasing reimbursement for services is how to maintain quality care. There is a looming shortage of oncologists that is forecast to impact cancer care significantly in the next few years (Erikson et al., 2007). Advanced practitioners are one solution to help fill the gaps. For this to come to fruition, the delivery of oncology care will need to be well organized, efficient, and quality driven.

In an effort to address current disparities in the standards of care delivered by oncology practices, evidence-based guidelines with disease-specific metrics have been created. A collaborative effort in 2005 between the American Society of Clinical Oncology (ASCO) and the National Comprehensive Cancer Network (NCCN) created the Standard Practice Guidelines for breast and colorectal cancers. They based the guidelines on metrics for these cancers developed by the National Initiative for Cancer Care Quality (NICCQ) and the NCCN measures (Desch et al., 2008). Three measures were established for breast cancer and four were established for colorectal cancer (Desch et al., 2008). In 2005, ASCO also developed the Quality Oncology Practice Initiative (QOPI), which includes multiple measures of quality cancer care (Neuss et al., 2013). These and other professional oncology organizations are listed in the Table.

**Table 1:**
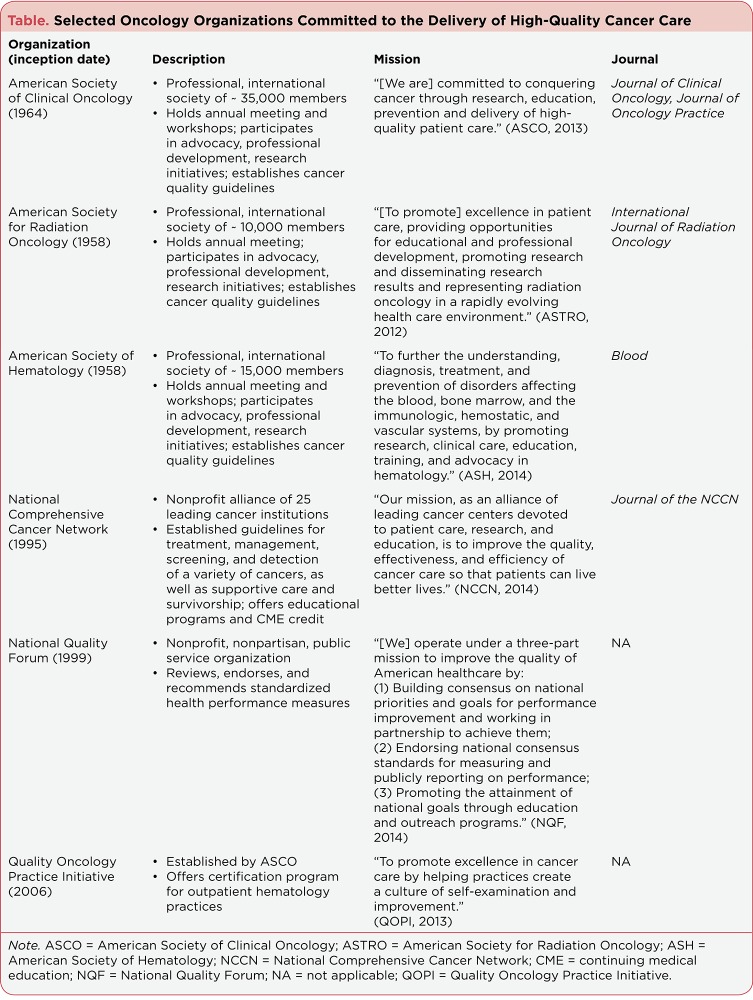
Selected Oncology Organizations Committed to the Delivery of High-Quality Cancer Care

Outside of Medicare, other insurance companies are also influencing oncology care. For example, as of July 2014, WellPoint (one of the nation’s largest health benefit companies) partnered with AIM Specialty Health to initiate the Cancer Care Quality Program (AIM, 2014). The goal of this initiative is to offer monetary incentives to oncologists who prescribe treatments that comply with the WellPoint Cancer Treatment Pathways (AIM, 2014). WellPoint states that these pathways were developed using evidence based guidelines and take into account "clinical efficacy, side effects, strength of national guideline recommendations, and cost" (AIM, 2014), The initial pathways include breast, lung, and colorectal cancer, but pathways for other malignancies are planned to be developed throughout 2014 and 2015. This provides an example of how insurance companies are providing incentives to standardize quality of oncology care as well as to rein in cost of these expensive therapies.

## IMPLICATIONS FOR THE ADVANCED PRACTITIONER

In the changing landscape of health care, the utilization of metrics is becoming an influential way to monitor and emphasize quality care. It is imperative for advanced practitioners (APs) in oncology to consider using metrics within their practices as a way to improve their quality of care, demonstrate clinical expertise, and quantify their contributions. Advanced practitioners should stay informed and engaged in the development, monitoring, and use of metrics pertinent to their practice.

Before incorporating metrics into practice, APs should first identify quality standards they wish to achieve, select appropriate evidence-based metrics, and then measure the outcomes. Advanced practitioners developing a metric profile for their practices should reference their specialty organizations, such as those listed in the Table. These and other organizations have developed evidence-based metric recommendations to help guide oncology practices. Along with other insurers, CMS may further influence the metrics chosen to measure and follow. It should be noted that the types of metrics used for each practice will vary; however, measures of quality and patient safety should always be included.

## CONCLUSIONS

Although reimbursement has not yet been tied directly to the quality of care delivered by the individual AP, this is highly probable in the future. By becoming active participants in establishing metrics within their own scope of practice and institutions, APs have the opportunity to influence quality standards as individual clinicians and for their professions as a whole.

**Acknowledgment**

We would like to acknowledge Regina Cunningham, PhD, RN, AOCN®, FAAN, for her leadership and direction; Genevieve Hollis, MSN, CRNP, AOCNP®, for her guidance and participation; and Jennie Greco Lattimer, MSN, ANP-BC, AOCN®, for her contributions to this project.
